# Content, controls, audience: contextualizing de-identification as a novel approach to enabling cross-sectoral data linkages

**DOI:** 10.23889/ijpds.v9i5.2847

**Published:** 2024-09-18

**Authors:** Rosario Cartagena, Michael Smith, Kathy Li, Ash-Lei Lewandoski

**Affiliations:** 1 ICES

## Background

Canadian privacy laws often distinguish personal health information (PHI) from non-health data. A recent legislative designation permits the Institute for Clinical Evaluative Sciences (ICES) cross-sectoral data linkages and analyses of health and non-health data but requires a discrete environment for each data type and only one environment to conduct research and analytics. It further stipulates that only de-identified, non-health data may enter the PHI environment where research and analytics are conducted, an added complication since data linkages necessarily require some identifiability. In order to consolidate its health and non-health data, then, ICES requires novel approaches to data governance and de-identification.

## Objective

To establish a comprehensive data governance framework that optimizes cross-sectoral data linkages and classifies identifiability as existing on a spectrum dependent on content, controls and audience.

## Approach

Operating under legislative and regulatory definitions of de-identification that contemplate what is “reasonably foreseeable in the circumstances,” ICES is working with its privacy regulator to create a data governance framework and de-identification guidelines for its health and non-health data.

## Results

While still awaiting regulatory approval, ICES will be a first-of-its-kind data integration unit whose data governance model is formally approved by its privacy regulator and an industry leader in novel approaches to de-identification and cross-sectoral data linkages.

## Conclusions

Identifiability is determined by context, and data may be simultaneously identifiable and de-identified, depending on its contextual factors.

## Implications

ICES’ approach to data governance and de-identification can offer important insights to other research and analytics-based organizations facing legislative barriers to cross-sectoral data linkages.

